# Occupational lung disease: A narrative review of lung conditions from the workplace

**DOI:** 10.1016/j.amsu.2021.102245

**Published:** 2021-03-23

**Authors:** Mohd Iskandar Jumat, Firdaus Hayati, Syed Sharizman Syed Abdul Rahim, Sahipudin Saupin, Khamisah Awang Lukman, Mohammad Saffree Jeffree, Helen Benedict Lasimbang, Fairrul Kadir

**Affiliations:** a Department of Community and Family Medicine, Faculty of Medicine and Health Sciences, Kota Kinabalu, Sabah, Malaysia; b Department of Surgery, Faculty of Medicine and Health Sciences, Kota Kinabalu, Sabah, Malaysia; c Department of Reproductive Health, Faculty of Medicine and Health Sciences, Kota Kinabalu, Sabah, Malaysia; d Department of Medicine, Faculty of Medicine and Health Sciences, Kota Kinabalu, Sabah, Malaysia

**Keywords:** Occupational lung disease, Silicosis, Asbestosis

## Abstract

Occupational lung diseases are lung conditions caused or made worse by materials when a person is exposed to a workplace. The diagnosis of an occupational disease is important for workers' decision to continue work and for their eligibility under compensation programmes. We revisit the existing lung diseases that are closely associated with the occupation at the workplace namely occupational asthma, silicosis, black lung disease, farmers’ lung disease, asbestos-linked disease, and hypersensitivity pneumonitis. Occupational lung diseases contribute toward global health and economic impacts. Prevention and control of occupational lung diseases require a collaborative effort among employers, workers, occupational physicians, pulmonary physicians, industrial hygienists, and members from other disciplines.

## Highlights

• Various occupational lung diseases can occur at workplace.
• The diagnosis is important for workers’ decision to continue working and the eligibility for compensation programmes.
• Prevention of occupational lung diseases requires a collaborative effort among employers, workers, occupational safety officers and pulmonary physicians.

## 1 Introduction

Occupational lung diseases or work-related lung diseases are lung conditions caused or made worse by materials when a person is exposed to irritants in the workplace. The diagnosis of an occupational disease is important as it has further implications on workers' or patients' decision to continue their work or not, and on their eligibility under compensation programmes and other benefits [1]. Occupational lung disease needs to be evaluated for respiratory complaints to highlight some simple and important questions, such as the patient's current and past occupations, home environment, hobbies, the potential for second-hand or para-occupational exposure, history of present illness, and timing onset of symptoms. In this review, some of the commonly reported occupational lung diseases will be discussed, such as occupational asthma, silicosis, black lung disease, asbestos-linked disease, and farmers' lung disease.

There a worrying increasing trend of occupational lung diseases reported in Malaysia. A study by Perakas Rav et al. among quarry workers in Malaysia had shown that respiratory problems are very closely related to cement dust exposure among those workers [2]. He also found that there was a marked reduction in lung function among those workers at the cement manufacturing plant who were exposed to the cement dust in high concentration. This was explained by another study by Amran et al. which also conducted at quarries in Malaysia which showed that respirable crystalline silica exposure levels were very significant above permissible exposure level (PEL) at all the quarries under his study [3]. Nurul et al. also found that the average trace metal dust concentration averaged over 8 h working period for chromium and cobalt in all plants under her study which above PEL in a steel factory situated at the eastern coast of peninsular Malaysia [4]. A study by Djoharnis et al. showed that respiratory disorders symptoms such as cough and excessive phlegm production in the morning, and difficulty in breathing were markedly higher among domestic waste collectors compared to administrative workers [5]. These lead to an increasing number of cases reported yearly to the Department of Occupational, Safety and Health (DOSH) Malaysia on Occupational Lung Diseases, for example, an increasing from 86 cases in 2015 to 150 cases in 2016 [2].

## 2 Occupational asthma

Asthma in the workplace can be defined as occupational asthma due to conditions in a particular work environment; work-aggravated asthma is caused by irritants or physical stimuli in the workplace [6]. Occupational asthma may have latency periods, which may develop after exposure ranging from few weeks to several years, while occupational asthma without latency periods follows exposure to high concentrations of irritant gases, fumes, or chemicals on one or several occasions [7]. Agents causing occupational asthma with latency consist of a wide range of materials from natural to synthetic chemicals or processes [8]. These agents can also be subdivided into IgE-dependent and IgE-independent, with each group causing distinctive clinical presentations. For instance, common agents that can induce occupational asthma without latency are chlorine and ammonia. A majority of high-molecular-weight compounds (≥5000 Da) induce asthma by producing IgE antibodies, and some low-molecular-weight compounds (<5 kDa), such as anhydrides and platinum salts, act as haptens and induce specific IgE antibodies by combining with body protein [9,10]. This will then give rise to a cascade of inflammatory events by activating inflammatory cells and releasing preformed and newly formed inflammatory mediators, which play a pivotal role in the inflammatory process.

Patients with occupational asthma exhibit bronchial hyperresponsiveness, which may decrease with time away from exposure, and increase with re-exposure to sensitising agents [11]. Asthmatic reactions can be divided into different stages based on specific inhalation challenge tests, which are isolated early, isolated late, biphasic, or continuous asthma reactions [12]. Isolated early asthmatic reactions occur within a few minutes after the inhalation challenge test, reach maximal intensity within 30 min, and end within 60–90 min. In contrast, isolated late asthmatic reactions occur 4–6 h after the inhalation challenge test, reach maximal intensity within 8–10 h, and end after 24–48 h. For instance, the biphasic reaction is an early reaction with spontaneous recovery, followed by a late reaction. On the other hand, a continuous asthmatic reaction has no remission between the early and late phases. In addition, atypical reactions that start 2 h after a challenge test and last for a few hours have also been defined [13]. In general, IgE-dependent agents induce isolated early or biphasic asthmatic reactions, while IgE-independent agents induce isolated late, biphasic, or atypical asthmatic reactions. Occupational asthma induced by IgE-dependent agents is similar to allergic asthma that is unrelated to work.

The symptoms of occupational asthma are the same with those of other asthma forms, where the airway-wall thickness increases because of the accumulation of inflammatory cells (mostly eosinophils), oedema, hypertrophy of smooth muscle, subepithelial fibrosis, and obstruction of the airway lumen by exudate or mucus. These are also found in patients who have been reported to die from isocyanate-induced asthma and occupational asthma [[14], [15], [16]]. Occupational asthma must be diagnosed with both establishments of a relationship between work and asthma. Occupational asthma caused by IgE-dependent agents can be diagnosed with a skin test or a blood test. Sensitisation and pharmacologically induced bronchial hyperresponsiveness can be observed with an 80% likelihood of immediate asthmatic reaction if a patient is challenged with the same occupational allergen in the laboratory [17]. People should be exposed to gradually increasing doses to avoid severe or irritant reactions [18]. If incompatible agents are used, or if a person has been away for an extended period and his/her bronchial responsiveness returns to normal, it can cause a false-negative result in a specific challenge test. Another approach is by monitoring patients' peak expiratory flow for two weeks while patients are at work, and another for two weeks while patients are away from work [19]. Although a good association has been found between peak expiratory flow monitoring and specific challenge tests, this diagnostic approach has been criticised because it depends heavily on patients’ cooperation, reliable performance, and recording correct readings [20,21]. The ideal treatment for occupational asthma patients with a latency period is the removal from exposure. Pharmacologic treatment of patients with occupational asthma is similar to treating patients with other forms of asthma.

## 3 Silicosis

Silicosis is caused by the inhalation of crystalline silicon dioxide or silica, which occurs naturally in rocks, sand, and other building materials such as concrete, ceramics, bricks, and tiles [22]. It shows latency for approximately 10–30 years, or develops earlier in a person exposed to high quantities of fine silica dust over a relatively short period, usually months, in a process known as accelerated silicosis [[22], [23], [24], [25], [26]]. Workers involved with mining, quarrying, drilling, foundries, ceramics, and sandblasting industries are at high risk to develop silicosis [22,24]. When inhaled, silica particles usually measuring 1–2 mm are ingested by the alveolar macrophages (AM) in alveoli. The cytotoxic effect of silica causes macrophage death, followed by the release of inflammatory cytokines and other substances that induce proliferation of fibroblasts. These fibroblasts form hyalinised nodules composed of concentric layers of collagen and silica surrounded by a fibrous capsule. When crystalline silica in these nodules’ periphery is induced, a further fibrotic response of new silicotic nodules forms and ultimately leads to complicated silicosis or progressive massive fibrosis (PMF). Lymph nodes become involved when silica-containing macrophages reach the hila and mediastinum [27].

Silicosis can have different forms, ranging from acute to chronic. Electron microscopy reveals the lungs of acutely infected silicosis patients to present hypertrophic type II pneumocytes lining the alveoli, which may produce excessive amounts of proteinaceous materials and surfactant protein. It may then become filled with protein-containing materials [28]. Other than that, excessive radicals also play a role in the development of acute silicosis. Freshly fractured silica may generate a stronger inflammatory response as they contain a higher proportion of free radicals than that of intact silica and contain abundant cleaved particle surface where surface ROS (reactive oxygen species) tend to form [[29], [30], [31]]. These free radicals may alter transcription factors that lead to cell and/or DNA damage [32]. In chronic silicosis, an inflammatory storm within the alveoli leads to pulmonary fibrosis. This results as silica particles are not efficiently cleared from the lung by alveolar macrophages, thus becoming damaged. This may induce and cause the release of ROS, RNS, and excess free radicals [33]. This will be followed by the release of transcription factors (NFκB and activator protein 1) which trigger the production of inflammatory cytokines (TNF-α, IL1β and IL6), protease, arachidonic acid metabolites (leukotriene B4, prostaglandin E2), and chemokines (IL8 macrophage inflammatory protein MIP2, MIP1a, MIP1B [[33], [34], [35], [36]]. When an alveolar macrophage containing silica dies, it releases silica particles and are re-engulfed by other alveolar macrophages; thus, repeating the injury cycle [31]. Neutrophils and lymphocytes recruited to the lesion area result in further inflammation. Chronic silicosis is the usual form which develops after a decade of continuous exposure to a high concentration of silica dust. Symptoms are usually not developed, and radiographic confirmation is required for diagnosis, revealing nodular lesions located at upper lobes present on chest radiograph [37]. The patient may also present a condition known as accelerated silicosis with a history of a shorter time course of exposure to silica, ranging from 5 to 15 years [38]. Accelerated silicosis has been associated with a variety of autoimmune disorders. Acute silicosis has a low rate of occurrence, and patients diagnosed with acute silicosis may report substantial occupational exposure to silica over a short period. Acute silicosis patients may present symptoms of dyspnoea, fatigue, weight loss, fever, and pleuritic pain.

Several treatments have been used to decrease pulmonary inflammation response to silica, but no effective treatment is available. Corticosteroid and aluminium citrate have been used to treat silicosis. Corticosteroid was reported to have improved inflammatory bronchoalveolar lavage and pulmonary functions [39,40]. Aluminium citrate treatment also shows symptomatic improvements by coating silica particles within the lung; thus, reducing their solubility [41]. Polyvinylpyridine-N-oxide (PVPNO) has also shown to concentrate silica particles inside the cell by acting as a hydrogen acceptor and coat surface of silica particles; thus, reducing silica toxicity [42]. PVPNO treatment have managed to improve patients’ pulmonary capabilities and the course of disease in patients. PVPNO also decreases the generation of ROS and silica-induced DNA damage by selectively blunting the active sites at the particle surface. Other therapies have been evaluated, including the use of alveolar macrophage inhibitor and monoclonal antibodies directed against IL1 [43].

## 4 Black lung disease

Coal Worker's Pneumoconiosis (Black Lung Disease) results from the exposure to washed coal or mixed dust consisting of coal, kaolin, mica, and silica [23,25]. Although the exact mechanism and degree of lung injury are less clear, it is thought that the quantity and size of coal dust particles inhaled are likely the most causative factors [44]. CWP is characterised histologically by aggregates of coal dust and fibroblasts, which comprise the coal macule lacking hyalinisation and laminated collagen. Coal macules accumulate around the respiratory bronchioles, causing bronchiectasis. CWP can be distinguished from silicosis by the presence of coal dust and the absence of silicotic nodules.

## 5 Asbestos-linked disease

Asbestos is a naturally occurring fibrous silicate material used in construction and several manufacturing industries such as brake linings and pads, tiles, bricks, insulation materials, and lining of furnace ovens. Asbestos can be categorised into two serpentine groups and amphibole fibres. Serpentines (e.g., chrysotile and amphibole) display the curly stranded structure, whereas amphiboles (e.g., crocidolite, amosite, tremolite, and others) are straight, rod-like fibres. All of these asbestos fibres have been linked to carcinoma, mesothelioma, and lung fibrosis [45]. Asbestos industrial production began in the 1850s; however, by the mid-20th Century, it was evident that asbestos exposure increases the risk of non-malignant inflammatory (pleural effusion, pleural plaques, rounded atelectasis, and asbestosis) and malignant (mesothelioma and bronchogenic carcinoma) pulmonary diseases [[45], [46], [47], [48], [49]]. At least 40 countries, including the US, have banned or severely restricted asbestos use [50]. It is also suggested that asbestos exposure increases the risk of lung cancer [[51], [52], [53], [54], [55], [56], [57]]. The toxic effect of asbestos depends on the initial exposure and physical properties of different asbestos fibres [47,48,58,59]. After asbestos inhalation, alveolar epithelial cells (AEC) and alveolar macrophages (AM) internalise the fibres, which cause oxidative stress to the lungs. Reactive oxygen species (ROS) and reactive nitrogen species (RNS) produce the crucial toxicity of asbestos [48,53,59]. To date, there are no secondary preventive or curative mechanisms for disease-related asbestos. Treatment for lung cancer linked with asbestos exposure is the same as treatment for other lung cancers.

## 6 Farmers’ lung disease

Hypersensitivity pneumonitis (HP) or extrinsic allergic alveolitis was first described in farmers and bird breeders, resulting in terms such as “farmers' lung” and “bird fancier's lung.” It is caused by the diffusion of granulomatous interstitial involving lungs and terminal airways. Farmers' lung disease results from repeated inhalation of antigenic organic and low molecular weight inorganic particles [23,[60], [61], [62], [63]]. Isocyanates (paint sprays), plastics, *Mycobacterium avium* complex (metalworking fluids), *Aspergillus,* and thermophilic actinomyces are the common antigens of HP [64]. HP can be grouped into acute, subacute, and chronic forms, based on clinical and radiological evidence at diagnosis [23,60,63]. Acute conditions occur after exposure to a high concentration of antigens over a short period, with symptoms appearing from 4 to 8 h and tend to resolve quickly. The acute patient shows nonspecific symptoms such as malaise, fever, and dry cough. At the same time, the subacute form occurs after continuous moderate exposure to antigens. Symptoms are malaise, fever, asthenia, anorexia, dyspnoea, and non-productive cough. Chronic patients develop these after exposure to lower antigen levels, but for more extended periods or in untreated acute and subacute patients. Symptoms are dyspnoea with a dry cough, and physical examination reveals digital clubbing and dry crackles on auscultation [65].

Antibodies, particularly IgG, recognise antigens and form antigen-antibody complexes. This Th1 immune response by cytotoxic CD8^+^ lymphocytes is also involved in lymphocyte alveolitis and granulomas formation; study showed their involvement in the pathogenesis of FLD and diagnostic marker of disease [[66], [67], [68]]. Diagnosis of Farmers’ Lung Disease includes clinical laboratory testing, skin prick testing, bronchial challenge testing, radiology, respiratory function, specific inhalation challenge, bronchoscopy, transbronchial biopsy, cryobiopsy, and surgical lung biopsy. Currently, the best way to treat FLD is by avoiding exposure to antigens. Glucocorticosteroids have been shown to accelerate recovery in acute forms, but it does not affect the chronic form [67,69]. There are several ways to prevent FLD, which are withdrawal of the patient from the farming environment, development of the new drying hay technique, and the use of respiratory protective equipment.

## 7 Conclusion

Occupational lung diseases are related to matters or particles inhaled from the workplace environment, including chemicals, dust, and proteins. They contribute toward global health and economic impacts worldwide including Malaysia. Prevention and control of occupational lung diseases require a collaborative effort among employers, workers, occupational physicians, pulmonary physicians, industrial hygienists, and members from other disciplines.

## Sources of funding

This write-up was supported financially by the 10.13039/501100006242Universiti Malaysia Sabah, Kota Kinabalu, Sabah, Malaysia under a special grant of SDK2018-0052.

## Ethical approval

The research study was approved under the Universiti Malaysia Sabah Medical Research Ethic Committee with the approval number of JKEtika 3/18 (9).

## Consent

Consents are not required.

## Author contribution

MIJ wrote the initial manuscript draft and involved in literature search.

FH provided the study design and final review.

SSSAR analysed the data and supervised the study progress. SS provided the expert opinion and supervised the study progress.

KAL provided the expert opinion.

MSJ involved in manuscript review.

HBL provided the expert opinion.

FK involved in manuscript review.

## Trial registry number

Not related.

## Guarantor

Firdaus Hayati.

## Declaration of competing interest

No potential conflicts of interest relevant to this article were reported.
